# An 8-channel Tx/Rx dipole array combined with 16 Rx loops for high-resolution functional cardiac imaging at 7 T

**DOI:** 10.1007/s10334-017-0665-5

**Published:** 2017-11-24

**Authors:** Bart R. Steensma, Ingmar J. Voogt, Tim Leiner, Peter R. Luijten, Jesse Habets, Dennis W. J. Klomp, Cornelis A. T. van den Berg, Alexander J. E. Raaijmakers

**Affiliations:** 1 Department of Radiology, University Medical Center Utrecht, Utrecht, The Netherlands; 2 Department of Radiotherapy, University Medical Center Utrecht, Utrecht, The Netherlands; 3 Department of Biomedical Engineering, Eindhoven University of Technology, Eindhoven, The Netherlands

**Keywords:** Ultrahigh field, Dipole antennas, RF coil arrays, Cardiac imaging

## Abstract

**Objective:**

To demonstrate imaging performance for cardiac MR imaging at 7 T using a coil array of 8 transmit/receive dipole antennas and 16 receive loops.

**Materials and methods:**

An 8-channel dipole array was extended by adding 16 receive-only loops. Average power constraints were determined by electromagnetic simulations. Cine imaging was performed on eight healthy subjects. Geometrical factor (g-factor) maps were calculated to assess acceleration performance. Signal-to-noise ratio (SNR)-scaled images were reconstructed for different combinations of receive channels, to demonstrate the SNR benefits of combining loops and dipoles.

**Results:**

The overall image quality of the cardiac functional images was rated a 2.6 on a 4-point scale by two experienced radiologists. Imaging results at different acceleration factors demonstrate that acceleration factors up to 6 could be obtained while keeping the average g-factor below 1.27. SNR maps demonstrate that combining loops and dipoles provides a more than 50% enhancement of the SNR in the heart, compared to a situation where only loops or dipoles are used.

**Conclusion:**

This work demonstrates the performance of a combined loop/dipole array for cardiac imaging at 7 T. With this array, acceleration factors of 6 are possible without increasing the average g-factor in the heart beyond 1.27. Combining loops and dipoles in receive mode enhances the SNR compared to receiving with loops or dipoles only.

**Electronic supplementary material:**

The online version of this article (10.1007/s10334-017-0665-5) contains supplementary material, which is available to authorized users.

## Introduction

Cardiac magnetic resonance imaging (CMRI) at ultrahigh field (UHF, *B*_0_ ≥ 7.0 T) strengths holds promise for several clinical applications. Coronary artery imaging has been applied at UHF strength and is reported to have higher a signal-to-noise ratio (SNR) and contrast-to-noise ratio (CNR) compared to 3 T [1, 2]. Applications such as functional imaging and quantitative parameter mapping have also been demonstrated at 7 T [3–7]. However, UHF–CMRI is challenging due to UHF-inherent phenomena such as transmit field (*B*_1_^+^) and background field (*B*_0_) inhomogeneities and increased energy deposition [4, 8]. Recent advances in RF transmit coil array design have been utilized to enhance transmit efficiency and homogeneity while keeping the specific absorption rate (SAR) within the required limits [9–13]. Improved RF shimming and pulse design can be used for further improvements [14–16], and advances in RF modelling have led to the adoption of less conservative SAR limits [17, 18].

Developments in transmit array design have demonstrated the beneficial use of dipole antenna arrays for body imaging at 7 T [9, 10, 13, 19]. The use of fractionated dipole antennas can lead to lower SAR levels while maintaining transmit efficiency [9]. Conversely, for signal reception, current patterns corresponding to a combination of electric dipoles and magnetic dipoles yield the theoretical ultimate intrinsic SNR [20–22]. These current patterns correspond to a receive array composed of dipole antennas and loop coils.

Loop coils are commonly used as receive elements in cardiac imaging, often in combination with a body coil at 1.5 and 3 T, or in transmit/receive mode at 7 T [12, 23]. More recently, dipole antennas have been used as transmit/receive elements for cardiac imaging at 7 T [2, 10, 13]. We present a body array that consists of 8 fractionated dipole antennas in transmit/receive (Tx/Rx) mode and 16 loop coils in receive (Rx) mode [10, 11, 24] resulting in an 8-channel Tx/24-channel Rx array. This should provide SNR enhancement, while not having to extend the 8-channel transmit chain in our current multi-transmit system. The array is specifically adapted for cardiac imaging by modifying the shape of the elements to the torso to maintain full contact between the antenna elements and the tissue. Electromagnetic simulations have been used to assess safety limitations of this setup for cardiac imaging. Imaging performance is demonstrated for functional cine imaging and compared to the imaging performance reported for cardiac 7-T imaging in the literature [12, 13].

## Materials and methods

### Transmit/receive setup

**Fig. 1:**
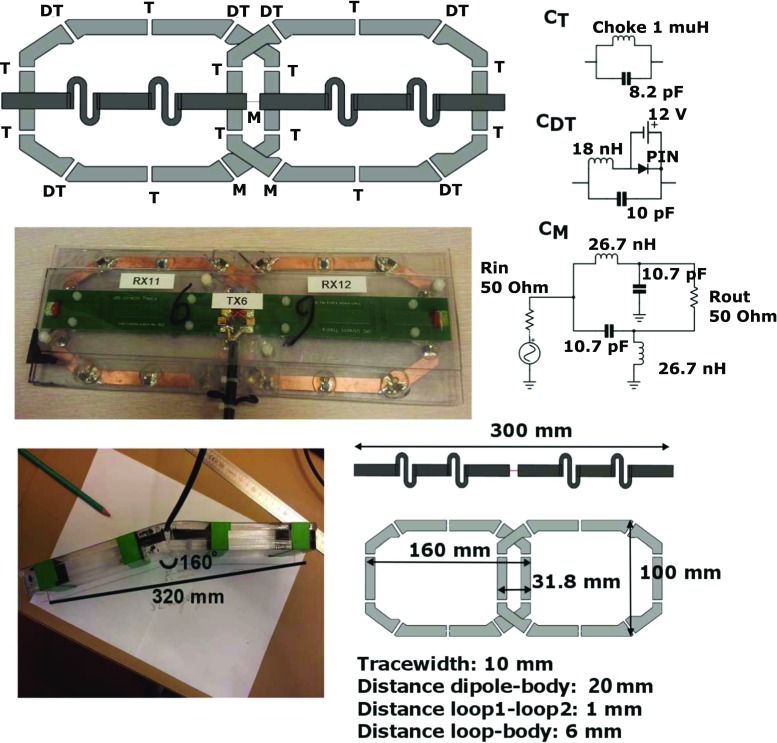
Schematic overview of a single-loop dipole element. **a** Shows a model of the loops and the dipoles and indicates the position of the tuning, detuning and matching circuitry. A lattice balun was used for matching both the loops and the dipoles. **b** Shows a photograph of a single-loop dipole element. **c** Shows the detuning and matching circuitry. **d** Shows one of the two elements that is adapted to the curvature of the chest, by bending both ends of a single element. **e** Shows the sizes of the loop and dipole elements

A custom-built 8-channel Tx/24-channel Rx setup was fabricated (Fig. 1). The setup consists of 8 building blocks composed of 8 fractionated dipole antennas (300-mm length) that were used for both transmitting and receiving. In each building block, two loop coils (160/100 mm, long/short axis) were positioned between the dipole and the subject along the longitudinal direction with overlap for decoupling. These 16 loop coils were used for receiving only [9, 11]. The dimensions of the loops and the dipoles are based on simulations on ideal loop and dipole size, which were done in references [9, 25].

The loop coils were kept at a 6-mm spacing from the body and the dipole was kept at a 20-mm distance from the body by a polycarbonate housing of each building block. The two medial anterior elements were bent in the middle and positioned at a fixed angle to maintain full contact with the chest. Each loop coil was detuned at three positions by PIN diodes. Preamplifier decoupling ensured high impedance at the cable connection point during reception. A lattice balun was used for impedance matching of both the dipole antennas and the loop coils. More details on geometry and circuitry are presented in Fig. 1. Reflection and coupling levels (S11 and S12) were determined to ensure good array performance as well as Qunloaded-to-Qloaded ratios for the loop coil elements.

### Electromagnetic modeling

The 8-channel Tx/24-channel Rx setup was modeled using the Sim4Life environment (Zurich Medtech, Zurich, Switzerland). Simulations were carried out on human models Duke and Ella [body mass index (BMI) 23.1 and 22 kg/m^2^], using a resolution of 1.5 × 1.5 × 1.5 mm^3^ to render the coil geometry and the nearby tissue, resulting in 13.862 × 10^6^ cells for Duke and 13.746 × 10^6^ cells for Ella [26]. Simulations were done on a graphic processor unit (GPU, NVIDIA GeForce GTX TITAN Black, NVIDIA, Santa Clara, CA, USA). [26]. The worst-case SAR was calculated as the maximum sum of the modulus of all quality matrix (Q-matrix) entries [18]. This value was used to derive average power limits for imaging applications, based on a 10-g-averaged SAR limit of 20 W/kg in the trunk for the first level controlled mode [27]. To validate the simulations, single-channel *B*_1_^+^ maps have been acquired for all transmit channels on a phantom filled with ethylene glycol and saline (*ε* = 34, *σ* = 0.4 S/m, 0.4 g/L saline) [9]. The dual refocusing angle acquisition mode (DREAM) *B*_1_^+^ mapping method was used to acquire the *B*_1_^+^ maps [28]. The imaging parameters of this sequence were as following: 2D acquisition, echo time (TE) = 1.57 ms, repetition time (TR) = 10 ms, field of view (FOV) = 320 × 400 × 30 mm^3^, in-plane resolution = (5 × 5) mm^2^, slice thickness = 30 mm, flip angle = 10°, pulse width = 0.17 ms, stimulated echo (STE) angle = 60°, pulse width = 1.01 ms, nominal *B*_1_^+^ = 16 μT, receive bandwidth = 4882.8 Hz, acquisition time = 11 s. A model of the phantom was imported from SolidWorks (SolidWorks, Dassault Systèmes SolidWorks Corp., MA, USA) into Sim4Life and used for simulations of the *B*_1_^+^ fields. An isotropic resolution of 1.5 × 1.5 × 1.5 mm^3^ was used for the full model, resulting in a total of 22.687 × 10^6^ cells.

### Cine imaging experiments

A 7-T Philips Achieva multi-transmit system with 8 × 2 kW RF amplifiers (Philips Healthcare, Best, The Netherlands) was used to scan 8 healthy volunteers [7 males, 1 female, age 22–35, average BMI = 21.6 ± 1.14 kg/m^2^, minimum BMI = 20.1 kg/m^2^ and maximum BMI = 23.1 kg/m^2^]. The study was approved by the local medical ethics committee and all subjects signed informed consent prior to inclusion in the study. RF phase shimming was applied on three slices in the heart, in order to obtain the maximum average signal in the heart [29, 30]. For the phase shimming, low-flip-angle gradient echo images were acquired in three slices for every transmit channel, these images were combined to obtain maximum signal intensity averaged over three slices in the heart; the optimum phase settings were calculated with a numerical minimization in Matlab (Mathworks, Natick, MA, USA) [30]. The same phase settings were used for all three slices, while the amplitudes of the channels where all set equally. This procedure was done once for every volunteer, while the same phase settings were used for all views. Phase-only RF shimming was used for every acquisition in this work. The following imaging parameters were used for the low-flip-angle gradient echo images: 2D multislice acquisition (M2D), TE = 1.68 ms, TR = 24 ms, FOV = 309 × 522 × 60 mm^3^, in-plane resolution = (1.3 × 1.3) mm^2^, slice thickness = 20 mm, flip angle = 3°, turbo field echo factor (TFE-factor) = 15, receive bandwidth = 498.7 Hz, pulse width = 0.20 ms, nominal *B*_1_^+^ = 4 μT and acquisition time = 102.7 s. Subsequently, a 10-slice cine planning sequence in the transverse orientation was acquired during 5 breath-holds (*R* = 2). The obtained images were used for planning of cine cardiac imaging. Pseudo 2-chamber, pseudo 4-chamber, short-axis and 4-chamber (p2Ch, p4Ch, SAX and 4Ch, respectively) view images were acquired during breath-holds. The following imaging parameters were used for the cine imaging: TE = 2.7 ms, TR = 4.2 ms, FOV = 280 × 420 × 8 mm^3^, in-plane resolution = (1.3 × 1.3) mm^2^, slice thickness = 8 mm, flip angle = 9°, TFE-factor) = 10, receive bandwidth = 998.2 Hz, pulse width = 0.61 ms, nominal *B*_1_^+^ = 4 μT and acquisition time = 10 heartbeats/10 s on average, with 30 cardiac phases. Retrospective gating with electrocardiographic (ECG) pads and breath-hold triggering were used for motion compensation. For one volunteer, the 4Ch view acquisition was repeated at increasing resolutions (1.3 × 1.3 × 8 mm^3^, 1.1 × 1.1 × 2.5 mm^3^ and 0.75 × 0.75 × 1.75 mm^3^), using a sensitivity-encoding (SENSE) acceleration factor of R2 in the anterior–posterior (AP) direction. Acquisition times increased from 10 to 12 and 17 heartbeats, respectively.

### Cine image analysis

To obtain a measure of the overall image quality, the cine images of all eight volunteers were rated on a four-point scale by two experienced readers. Overall image quality, artifacts and noise where taken into account in this rating [23], where higher scores represent better image quality. The rating scale and scoring criteria are shown in detail in Table 1. Inter-observer agreement percentages and Cohen's kappa were used to calculate inter-observer variability for all ratings [31].

**Table 1:** Rating scale and scoring criteria for the functional cine images

Score	3	2	1	0
Artifacts	No artifacts	Minor artifacts (not impairing diagnostic quality)	Moderate artifacts (may partially impair diagnostic quality)	Major artifacts, not diagnostic images
Noise	No remarkable noise	Little noise (not impairing diagnostic quality)	Moderate noise (may partially impair diagnostic quality)	High noise level, not diagnostic
Overall image quality	Excellent	Good	Diagnosis may be limited	Poor, not diagnostic

### SNR and acceleration performance

In order to make a quantitative comparison to other literature, the imaging parameters used in [13] have been reproduced to the best of our ability. The following imaging parameters were used for the SNR analysis: TE = 3.8 ms, TR = 6.0 ms, FOV = 280 × 420 × 2.5 mm^3^, in-plane resolution = (1.1 × 1.1) mm^2^, slice thickness = 2.5 mm, flip angle = 20°, TFE-factor = 9, receive bandwidth = 998.2 Hz, pulse width = 3.6 ms, nominal *B*_1_^+^ 1.5 μT, acquisition time = 20 heartbeats/20 s on average, 28 cardiac phases, acceleration factor R = 2 and applied along the AP direction. This was done on three additional volunteers (2 males, 1 female, age 25–36, average BMI = 21.9 ± 1.12 kg/m^2^, minimum BMI = 20.01 kg/m^2^, maximum BMI = 23.03 kg/m^2^) in the 4Ch view and the SAX view. Phase shimming was done for all acquisitions on three transverse slices in the heart. To assess the SNR performance of the coil array, as well as the separate contributions of the loop and dipole elements, SNR-scaled images were reconstructed according to the method described by Robson et al. [32]. The mean SNR in the heart, and the CNR between the myocardium and the blood, defined as (SNR_blood_–SNR_myo_), was calculated for all three volunteers, according to [13].

To assess the acceleration performance of the array, the cine acquisitions were repeated on the same three volunteers, using sensitivity encoding (SENSE) with acceleration factors ranging from *R* = 2 to *R* = 6. Phase encoding was applied along the left–right (LR) direction for the 4Ch view images and along the feet/head (AP) direction for the SAX view images. Geometrical factor (g-factor) maps were reconstructed on the scanner using reconstruction software available on the Philips system (delayed reconstruction). The mean g-factor in the heart was calculated for all three volunteers.

## Results

### Transmit/receive setup

**Fig. 2:**
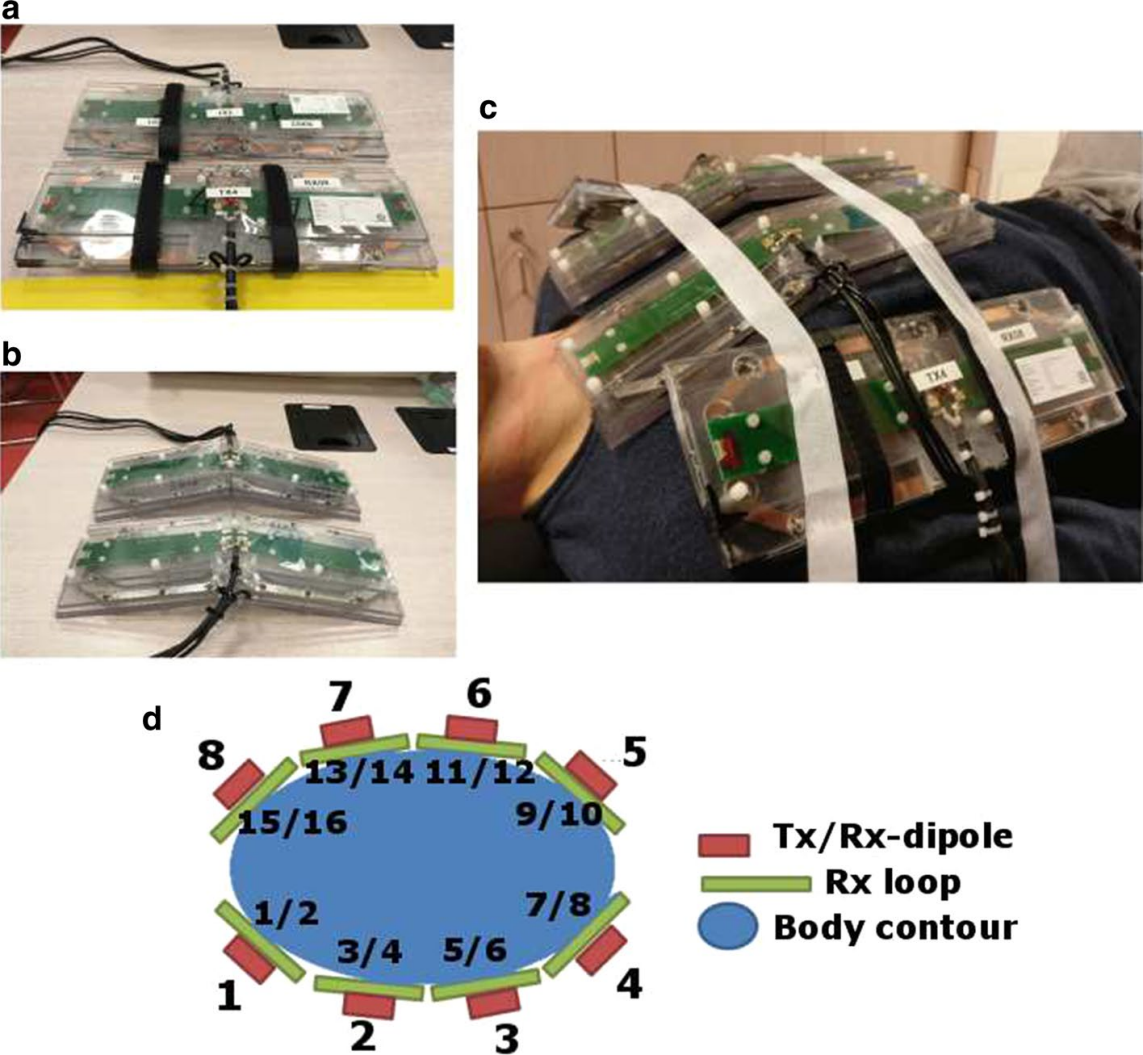
Schematic overview of the imaging setup. **a** Shows two elements consisting of a Tx/Rx antenna and two Rx loops. **b** Shows the two elements that are adapted to fit on the chest. **c** Shows a schematic drawing of the setup on a torso model. **d** Shows the transmit setup on a male volunteer. **e** Shows a noise covariance matrix on an exemplary volunteer

The elements were tuned and matched to obtain matching and decoupling values of −12 dB on the torso. Bench measurements show a *Q*_unloaded_-to-*Q*_loaded_ ratio of 140:11 for the loop elements. PIN diodes were used to detune the loops during RF transmission. Decoupling between the loops and the dipoles was improved from −12 to −18 dB or less after detuning of the receive loop. The geometry and circuitry of a single loop/dipole element is shown in Fig. 1. Figure 2 shows a schematic overview of the imaging setup on a volunteer.

### Electromagnetic modeling

**Fig. 3:**
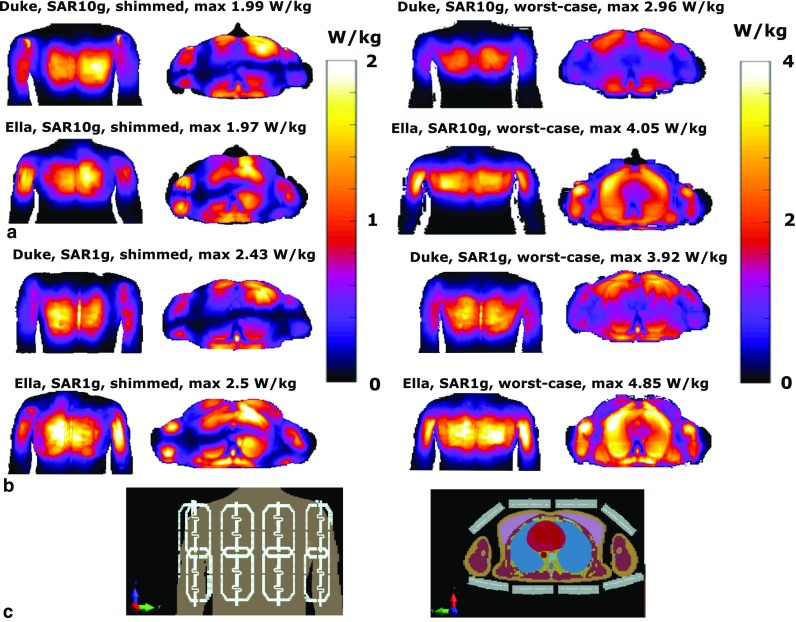
**a** Coronal and transverse maximum intensity projections of SAR_10g_ for the Duke and Ella model. Results are normalized to 1 W of input power for every input channel, using a total input power of 8 W. Input transmit phases are used to maximize average *B*_1_^+^ in the heart for the image on the left. The image on the right displays the worst-case SAR. **b** Shows a voxelized model of Duke from a frontal and transverse point of view

Figure 3 shows maximum intensity projections of 10-g-averaged SAR (SAR_10g_) and 1-g-averaged SAR (SAR_1g_) distributions for human models Duke and Ella. Phase-only shimming was used to optimize for maximum average *B*_1_^+^ in the heart and results are normalized to an average input power of 1.0 W per channel (8.0 W total). It was demonstrated that SAR_10g_ does not exceed 2.0 W/kg for both models using phase-shimmed transmit phases. The worst-case SAR_10g_ is calculated to be 4.05 W/kg for the Ella model, and 2.96 W/kg for the Duke model, using an input power of 1.0 W per channel (8.0 W total input power). Considering a maximum allowed SAR_10g_ of 20 W/kg in the first level controlled mode, the maximum average power limit is 20 W/4.05 = 4.92 W per channel based on these two models. The transmit phases for which the worst-case SAR values are obtained do not correspond to the transmit phases that maximize *B*_1_^+^ in the heart, so this value represents a conservative estimate of the required power constraint. However, with only two subjects investigated, inter-subject variability may still increase the maximum SAR value. A recent study has investigated these opposing effects for prostate imaging at 7 T [18, 33]. Based on these results, and including a safety margin of 20%, the average power limit in these experiments was set to 4.0 W per channel. The maximum worst-case SAR_1g_ that is calculated in simulations is 4.85 W/kg for a total input power of 1.0 W per channel. If the average power limit of 4.0 W/channel is applied, the local SAR limits are also not exceeded when using this small averaging volume.

**Fig. 4:**
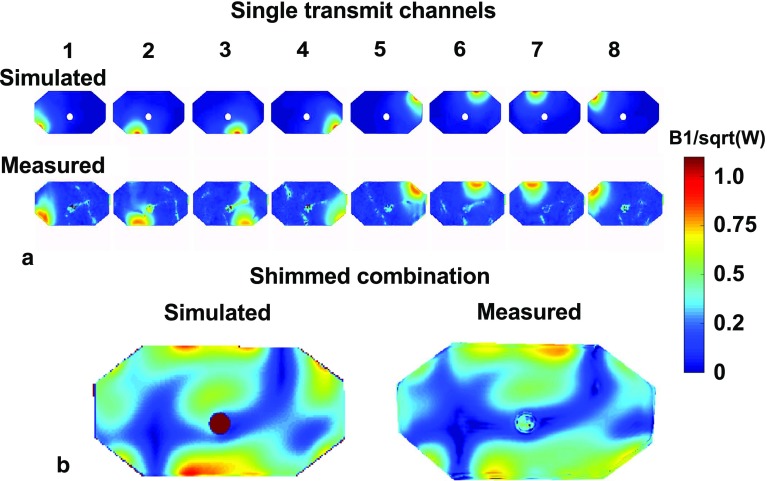
Phantom simulation setup. **a** Shows simulated (top) and measured (bottom) B_1_^+^ maps on the ethylene–glycol phantom. **b** Shows the same B_1_^+^ maps, now both combined using the same transmit phases

Figure 4 shows the simulation and measurement results on a phantom. The overall field patterns and the magnitude of the *B*_1_^+^ fields correspond qualitatively; however, some differences between simulations and measurements are to be noted. For all the single-transmit channels, minor differences between the simulated and measured field patterns can be discerned. In the shimmed combination, the field distribution in the center of the antenna corresponds well, but the measured transmit field at the bottom of the phantom has a lower intensity than the simulated field. The absence of highly intense peaks in the measured *B*_1_^+^ fields indicate that the loops are detuned well, and do not influence the transmit fields of the dipole antennas.

### Cine imaging experiments and analysis

**Fig. 5:**
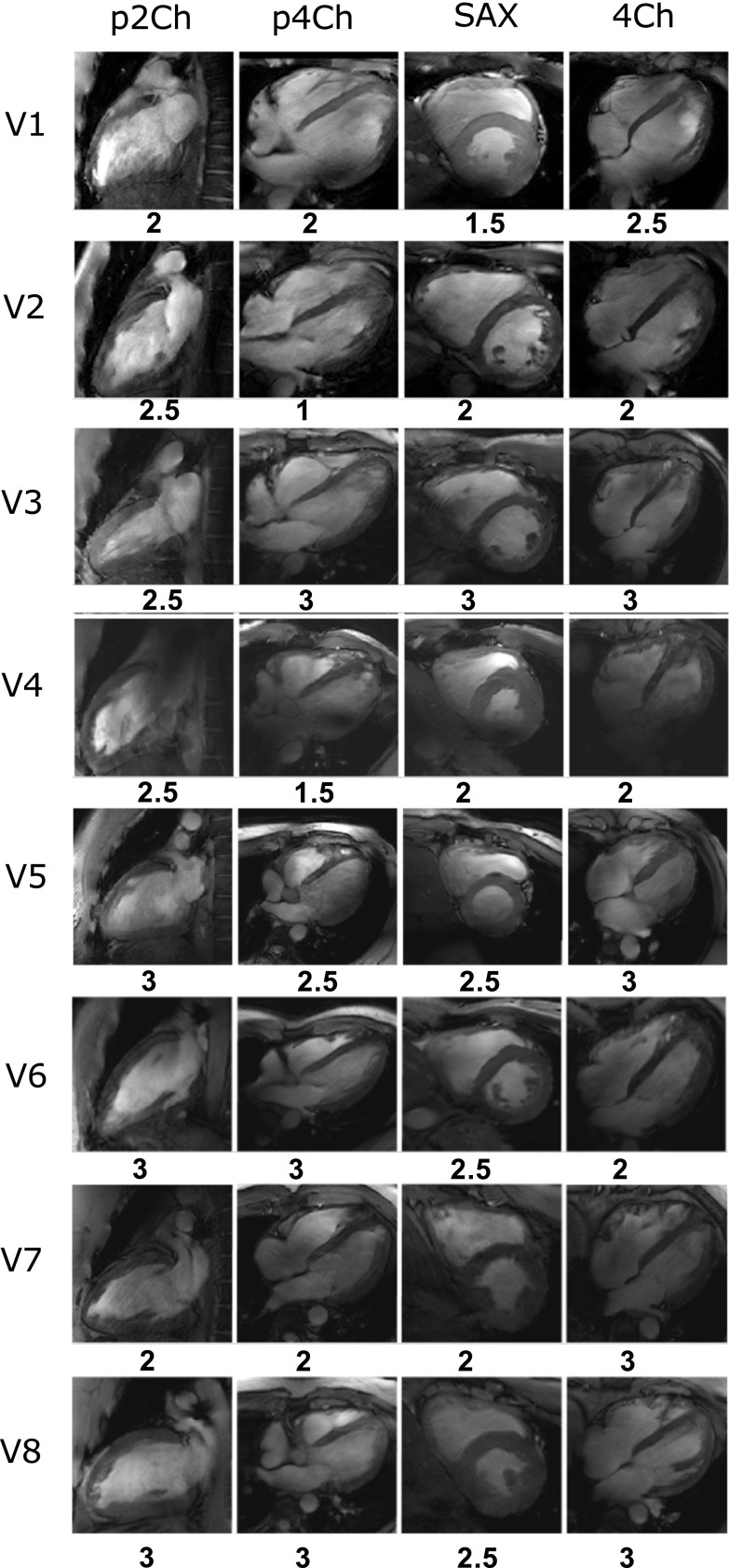
Pseudo two-chamber views (p2Ch), pseudo four-chamber views (p4Ch), short-axis views (SAX) and four-chamber views (4Ch) for eight volunteers. Phase-only shimming was applied to maximize the signal in three transverse slices for each individual volunteer. The same transmit phases were used for all acquisitions. All images were acquired with a resolution of 1.3 **×** 1.3 **×** 8 mm^3^, and an average scan time of 10 s. The overall image quality rating is displayed underneath each separate image

Figure 5 shows the imaging results of functional imaging for all eight subjects. Below each image, the average rating of overall image quality is reported. The image quality ratings are shown in Table 2 where 0 corresponds to poor image quality and 3 represents excellent image quality. A document with all the separate ratings is added as supplementary material. The overall image quality is rated between good and excellent (overall score 2.41). No remarkable noise is present in any of the images (overall score 3). Some of the images show artifacts (overall score 2.28), which can be recognized in Fig. 5 for volunteer 1 and 4. Inter-observer agreement was calculated for every view and criterion. The overall inter-observer agreement was 74%. Inter-observer variability was calculated using Cohen’s kappa, which is *κ* = 0.56 for all observations. This corresponds to a moderate agreement between both raters.

**Table 2:** Mean and standard deviation of image quality scores for all volunteers, for the different views and rating parameters

Parameter/view	2-chamber view	Pseudo 4-chamber view	Short-axis view	4-chamber view
Artifacts	2.50 ± 0.50 (75%)	2.25 ± 0.90 (50%)	2.50 ± 0.50 (37.5%)	2.6 ± 0.50 (62.5%)
Noise	3.00 ± 0.00 (100%)	3.00 ± 0.00 (100%)	3.00 ± 0.00 (100%)	3.00 ± 0.00 (100%)
Overall image quality	2.60 ± 0.50 (62.5%)	2.25 ± 0.90 (62.5%)	2.50 ± 0.50 (50%)	2.60 ± 0.50 (87.5%)

Inter-observer agreement for the different views and criteria are displayed below the scores

The average inter-observer agreement over all samples is 74%. Inter-agreement variability was also calculated using Cohen’s kappa, which was *κ* = 0.5221 over all observations

**Fig. 6:**
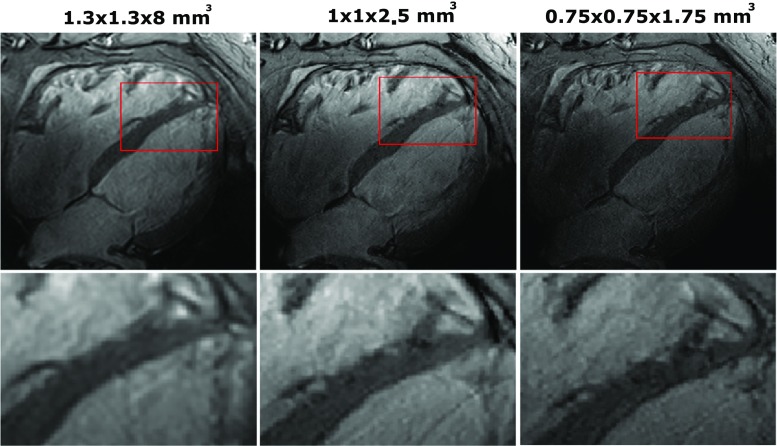
Four-chamber views using 2D cine acquisitions, at different spatial resolutions. All images were acquired with the same imaging parameters as the cine acquisition shown in Fig. 4, with an AP acceleration factor R2 and at different spatial resolutions. Acquisition time increased from 10 to 12 and 17 s. The bottom row shows the same images but zoomed in on the right cardiac chamber. At high resolution, improved depiction of the myocardial trabeculae in the right ventricular wall can be seen

Figure 6 shows the four-chamber view results of volunteer 6, using an acceleration factor of *R* = 2 and different resolutions. In general, as spatial resolution increases, SNR is reduced. However, due to the higher spatial resolution, more details can be recognized in the image, especially in the region close to the cardiac walls. The maximal resolution that was reached in this experiment on one volunteer is 0.75 × 0.75 × 1.75 mm^3^, which corresponds to a cubic resolution of 0.98 mm^3^. At this resolution, noise becomes more clearly present in the image, especially at locations far away from the transmit/receive elements.

### SNR and acceleration performance

**Fig. 7:**
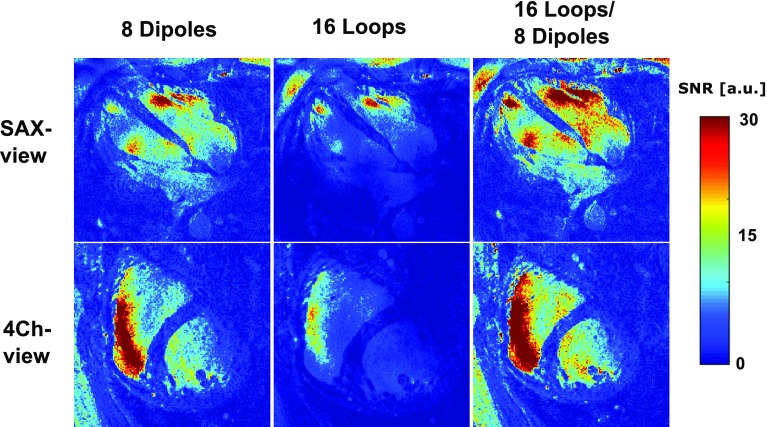
SNR-scaled images for a single volunteer in the SAX view and the 4Ch view. The separate contributions of the loop and dipole elements are displayed here. Phase shimming was applied on three transverse slices through the heart for all volunteers, and the same shim settings were used for all acquisitions. Images were acquired at a resolution of 1.1 **×** 1.1 **×** 2.5 mm^3^, at an average scan time of 20 s

Figure 7 shows SNR maps of a single volunteer, acquired in the 4Ch view and SAX view. The separate contributions of the loop and dipole elements are demonstrated in this image. It can be seen in both views that combining the loop and dipole elements leads to a remarkable enhancement of the SNR in the heart. Table 3 shows the SNR and CNR values in the heart for the different views, averaged over the three volunteers. When using all receive elements, the average SNR in the heart is 10.9 and 11.9 for the SAX view and the 4Ch view, respectively. The CNR_blood/myo_ for these views is 10.7 and 12.3. The SNR increases over two-fold when all elements are used, compared to the situation where only loops coils are used for receive. Compared to a situation where only dipoles are used in receive mode, the SNR increases 50% when all elements are used.

**Table 3:** Summarized results of the mean signal-to-noise ratio (SNR) and the mean blood/myocardium contrast-to-noise ratio (CNR_blood/myo_)

View	Setup	CNR (myocardium/blood)	SNR (whole heart)	Normalized SNR (whole heart; √Hz/ml)
SAX	16 loops	3.70 ± 2.70	2.70 ± 1.40	2.84 ± 1.5 × 1e4
4Ch	16 loops	2.50 ± 0.450	4.60 ± 0.80	4.80 ± 0.80 × 1e4
SAX	8 dipoles	9.07 ± 6.23	7.90 ± 3.60	8.30 ± 3.80 × 1e4
4Ch	8 dipoles	7.00 ± 1.01	7.60 ± 2.06	8.00 ± 2.20 × 1e4
SAX	16 loops/8 dipoles	12.70 ± 8.20	10.90 ± 4.70	11.50 ± 5.00 × 1e4
4Ch	16 loops/8 dipoles	10.30 ± 1.20	11.90 ± 1.60	12.50 ± 1.70 × 1e4

The contributions of the loop and dipole elements and the combined array are shown here for two views, averaged over three volunteers. SNR was normalized based on receive bandwidth and voxel volume

**Fig. 8:**
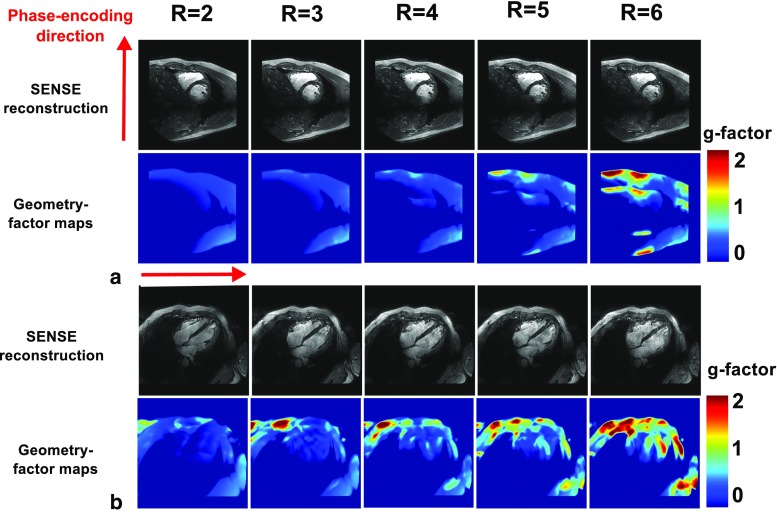
g-factor maps for different SENSE acceleration factors (*R* = 2 to *R* = 6) on a single volunteer in the SAX view and the 4Ch view. Increasing the acceleration factors increases the g-factor in the heart. Phase shimming was applied on three transverse slices through the heart for all volunteers; the same shim settings were used for all acquisitions. Images were acquired at a resolution of 1.1 **×** 1.1 **×** 2.5 mm^3^, with scantimes ranging from 20 to 7 s

Figure 8 shows reconstructed images and g-factor maps for a single volunteer, acquired in the SAX view and the 4ch view. As the acceleration factor is increased, the g-factor in the heart clearly increases, and noise becomes more present in the reconstructed images. However, even for an acceleration factor of *R* = 6, the anatomy of interest is still clearly visible. The average g-factor in the heart increases from 1.02 to 1.08 in the SAX view and from 1.04 to 1.27 when moving from an acceleration factor of *R* = 2 to *R* = 6. The maximum g-factor obtained in the SAX view is 2.00 at *R* = 6; in the 4Ch view, a g-factor of 3.1 is reached at *R* = 6 (see Table 4).

**Table 4:** g-Factors in the heart for increasing acceleration factors, with the average and maximum for three volunteers

		Acceleration factor				
		*R* = 2	*R* = 3	*R* = 4	*R* = 5	*R* = 6
View						
Phase-encoding direction		Average/maximum g-factor in the heart				
SAX						
AP		1.02/1.10	1.03/1.25	1.05/1.35	1.05/1.57	1.08/2.00
4Ch						
LR		1.04/1.43	1.06/1.61	1.10/1.65	1.19/2.40	1.27/3.10

## Discussion

This work demonstrates the potential of cardiac imaging at 7 T with a combined-loop dipole array. Several RF transmit setups have been developed for cardiac imaging, but the addition of 16 receive-only loops to a dipole array has not been demonstrated before. It is demonstrated here that combining 16 receive loops and 8 transceive dipoles leads to a 50% increase of SNR in the heart compared to a setup which used 8 transceive dipoles. SNR is sufficient to acquire detailed images at a high spatial resolution (1.1 × 1.1 × 2.5 mm^3^). When moving to a resolution of 0.75 × 0.75 × 1.75 mm^3^, a clear degradation of SNR is visible.

### Electromagnetic modeling

The worst-case SAR calculated here corresponds to the phase setting that yields the highest possible SAR value in the entire subject. This phase setting will not correspond to a realistic phase setting that is used for scanning. The safety simulations indicate that there is a large difference between the worst-case SAR and a SAR that corresponds to a realistic shim setting (The SAR corresponding to a realistic shim setting is 33 and 51% lower than the worst-case SAR, respectively, for Duke and Ella). While it is not likely that the worst-case SAR is obtained during a scan, this value was nevertheless used to set the upper limit of the total average power [33]. In other work [13], the total average power limits are derived based on peak SAR calculated in human models for fixed phase settings. These fixed phase settings are also used in the scan. Using this method leads to a total average power limit of 65 W, more than two-fold higher than the 8 × 4 = 32 W used in this work. This difference in the average power limit does not necessarily represent a difference in the efficiency of these two setups, but a difference in choices regarding transmit phase optimization and safety assessment.

Much effort has already been spent on matching SAR simulations with measurements as closely as possible [17, 18]. Future work into worst-case SAR estimations will very likely focus on deriving realistic drive settings for multiple human models and matching those to scan results, with the use of bi-directional couplers [33]. These methods have the potential to increase the average power limits, leading to faster cardiac exams at 7 T. Work focused on using nonlinear optimization of the transmit phases and amplitudes to reduce the maximum local SAR, or the use of SAR constraint RF pulses has not yet been applied in this work, and also have the potential to improve transmit efficiency [15, 17, 34–36].

Electromagnetic simulations were validated by comparing simulated to measured *B*_1_^+^ maps. Field measurements or MR thermometry are possible alternatives for safety validation, but are not treated within the scope of this paper. A comparison between simulated and measured transmit fields on a phantom correspond qualitatively. However, when comparing the results in detail, differences between the simulations and measurements are clearly visible. The qualitative correspondence between the results indicates that the antennas perform as modeled in the simulation, and it indicates that the loops are detuned properly. The results could be improved upon by exactly matching the simulation and measurement geometry by doing a computed tomography scan of the measurement setup and importing this in Sim4Life. Exactly matching the simulated and measured coupling parameters by using circuit co-simulations would be another way of improving the correspondence between simulations and measurements. This will be necessary for deriving SAR limits that closely match simulations for realistic drive settings, but it is not treated within the scope of this work.

### Cine imaging experiments and analysis

High-quality functional images were acquired for a total of eight volunteers. Image quality of the cine images has been scored by two experienced readers, leading to an average score of 2.4 (between good and excellent) for overall image quality. The rating shows that the diagnostic quality of the cine images is not at all affected by noise. The image quality scores are impacted by artifacts, which are most generally caused by a non-uniform excitation field. This is clearly present in volunteers 1 (SAX view) and 4 (p4Ch view), which is represented by the lower rating of the images. Although optimized RF transmit phase settings are calculated for every volunteer, it is clear that RF shimming methods that are used in this work do no suffice for every situation. The shimming method that is currently used maximizes the average signal in the heart, but does not necessarily provide a uniform signal. Additional RF calibrations can be used to enhance homogeneity of the signal, but this can also increase acquisition time and examination complexity [14, 16]. Future work will explore more advanced techniques to acquire homogeneous excitation fields combined with rapid calibration scans and procedures.

### SNR and acceleration performance

For SNR comparison, cine images have been acquired using scan parameters reproduced from literature to the best of our ability [13]. The flip angle of 30° could not be reproduced within a reasonable scan time. This is caused by different choices in setting the average power limits, as mentioned earlier in this discussion. The TR and TE that are reported by Oezerdem et al. (2.17 and 4.17 ms, respectively) was also not reproducible with our system and the other sequence parameters. The overall SNR values that we report at *R* = 2 are lower than for the two coil setups mentioned by Oezerdem et al. (11.9 compared to 29 for the 4Ch view, 10.9 compared to 29 for the SAX view). The CNR_blood/myo_ values that we report are comparable (10.3 compared to 11 for the 4Ch view, 9.9 compared to 9 for the SAX view). Because of the differences in acquisition methods, the results of this comparison should be interpreted with caution. An interesting comparison would be to scan both coil setups at the same imaging site; however, this will not be treated within the scope of this paper.

The average g-factor that we report at *R* = 6 is lower than the g-factors mentioned in literature (1.08 compared to 1.5 for the SAX view, 1.27 compared to 1.6 for the 4Ch view) [13]. As a result of the difference in MRI system vendor, SENSE is used in our work, while generalized autocalibrating partial parallel acquisition (GRAPPA) is used in [13]. Results show that the average g-factor remains low even up to an acceleration factor of 6. Although such accelerations may provide an insufficient SNR for many applications, these results show that in terms of encoding power of the array, it is possible.

The SNR maps presented here show that a setup which combines loops and dipoles in receive mode increases SNR in the heart by more than 50% compared to a setup where only loops or dipoles are used. This strong increase in SNR in not only caused by the receive sensitivity of the combined setup, but also relates to the improved acceleration that is gained my moving from 16 or 8 to 24 channels. Most setups that are used specifically for cardiac imaging make use of loop coils, while some setups use dipole antennas [9, 12, 13, 37–39]. This work shows that these kinds of setups can be improved by combining both elements.

## Conclusion

High-resolution cardiac cine imaging is demonstrated at 7 T using an 8-channel Tx/24-channel Rx array which combines loops and dipoles. The overall image quality of cine imaging results is rated a 2.6 on a 4-point scale by two experienced radiologists. Acceleration factors up to *R* = 6 can be used while the average g-factor in the heart does not exceed 1.27. Adding 16 receive-only loops to an 8-channel transceiver dipole array increases the SNR in the heart by more than 50% compare d to the use of dipoles only.

### Electronic supplementary material

Below is the link to the electronic supplementary material.

Supplementary material 1 (XLSX 12 kb)

Figure ESM2 simulated SNR for an 8-dipole/8-loop array and an 8-dipole/16-loop array. The dimensions of the phantom are 450 × 300 × 236 mm3, and the electrical properties are comparable to human tissue (*ε* = 34, *σ* = 0.4 S/m). The black ellipse represents a region comparable in size to the heart of virtual family model Duke (EPS 239 kb)

## Appendix A

Electromagnetic simulations on a phantom were done to assess the signal-to-noise performance of an 8-dipole/16-loop array, compared to an 8-dipole/8-loop array, as presented in [10]. The phantom had tissue-like properties (*ε* = 34, *σ* = 0.4 S/m), and the dimensions of the phantom were 450 × 300 × 236 mm^3^. Signal-to-noise ratio was calculated from the receive fields, which were normalized to input power and combined in a sum of magnitude sense, as in Ref. [10]. Figure 1 of the supplementary material shows the results of these simulations. The black ellipse represents a region with comparable dimensions to the heart of human model Duke. The sagittal and coronal slices clearly indicate that the array with two loops has a larger field of view. When looking at the SNR ratio in the central transverse slice, it can be seen that SNR increases only by a maximum of 10%, and even decreases closer to the coil elements. However, the central coronal and sagittal slices show that when moving away from the central slice, the SNR increases are strong (up to 50%) because of the larger field of view of the antennas. This is beneficial for cardiac imaging, which typically has a relatively large field of view.
